# Scaling prediction errors to reward variability benefits error-driven learning in humans

**DOI:** 10.1152/jn.00483.2015

**Published:** 2015-07-15

**Authors:** Kelly M. J. Diederen, Wolfram Schultz

**Affiliations:** Department of Physiology, Development, and Neuroscience, University of Cambridge, Cambridge, United Kingdom

**Keywords:** standard deviation, probability distribution, reinforcement learning, adaptation, risk

## Abstract

Effective error-driven learning requires individuals to adapt learning to environmental reward variability. The adaptive mechanism may involve decays in learning rate across subsequent trials, as shown previously, and rescaling of reward prediction errors. The present study investigated the influence of prediction error scaling and, in particular, the consequences for learning performance. Participants explicitly predicted reward magnitudes that were drawn from different probability distributions with specific standard deviations. By fitting the data with reinforcement learning models, we found scaling of prediction errors, in addition to the learning rate decay shown previously. Importantly, the prediction error scaling was closely related to learning performance, defined as accuracy in predicting the mean of reward distributions, across individual participants. In addition, participants who scaled prediction errors relative to standard deviation also presented with more similar performance for different standard deviations, indicating that increases in standard deviation did not substantially decrease “adapters'” accuracy in predicting the means of reward distributions. However, exaggerated scaling beyond the standard deviation resulted in impaired performance. Thus efficient adaptation makes learning more robust to changing variability.

an essential part of daily life is to predict which rewards will be available. Accurate estimation of future reward magnitude depends on our ability to learn the statistics of the environment. Rewards are not singular events with constant magnitude but are elements of probability distributions that fluctuate from one moment to the next. Even when fully informed about the anticipated mean, or expected value (EV), of probability distributions, we cannot predict the size of the next reward with certainty (O'Reilly 2013). Optimal performance can, however, be achieved by inferring the EV of distributions (Nassar et al. 2010).

The EV can be learned through errors in our predictions, i.e., reward prediction errors, as formalized in reinforcement learning models (Rescorla and Wagner 1972). In the Rescorla-Wagner reinforcement learning model, predictions are updated as a constant fraction of the prediction error, termed the learning rate. Rescorla-Wagner provides a powerful account of learning in nonvariable contexts where prediction errors converge to zero as predictions become more accurate (Schultz and Dickinson 2000). However, when outcomes fluctuate, predictions based on a constant learning rate can only become stable with low learning rates, resulting in slow learning (Payzan-LeNestour and Bossaerts 2011). Thus predictions can more rapidly become stable through dynamic learning rates that decrease as predictions become more accurate, as formalized in the Pearce-Hall reinforcement learning model (Pearce and Hall 1980) as well as in Bayesian accounts of learning (Cox 1946; Yu and Dayan 2005).

Importantly, learning may be further improved by scaling the prediction error relative to the expected fluctuation in reward value (Preuschoff and Bossaerts 2007). That is, a prediction error is more meaningful in contexts where rewards fluctuate less. Such scaling could facilitate earlier stability in predictions and similar learning for different degrees of reward variability, resulting in improved overall performance. In addition, adaptation to variability enables individuals to identify sudden changes in the outcome distribution (Nassar et al. 2010).

Thus far, it is unclear whether scaling of prediction errors relative to the variability of reward distributions results in improved performance, as predicted by learning models (Preuschoff and Bossaerts 2007). Increases in computational demands during prediction error scaling may, for instance, impede optimal deceleration of learning rates, resulting in suboptimal performance. In addition, although scaling of prediction errors relative to the variability in reward benefits performance, scaling with the standard deviation (SD) limits the power of the learning rate to update predictions. For instance, when a prediction error of 15 is divided by an SD of 15, the prediction can only be adjusted with 1 point (see Fig. 2*F*).

The main goal of this study was to investigate whether the scaling of prediction errors to reward variability (SD) in humans would be associated with superior performance. Participants were required to explicitly indicate the expected magnitude of upcoming rewards, drawn from probability distributions with different levels of variability. After each prediction, participants received a reward, eliciting trial-by-trial reward prediction errors. Participants' payoff depended on the points drawn by the computer, incentivizing them to treat the points as actual rewards. Performance was increased for gradual decreases in learning rates and scaling of prediction errors relative to, but smaller than, the SD. In addition, the individual degree of adaptation was predictive of the stability in performance across SDs, thus suggesting that adaptation made learning more robust to changing variability.

## MATERIALS AND METHODS

### 

#### Participants.

Thirty-one healthy volunteers (16 men, 15 women) were recruited through local advertisements. Participants were between 18 and 33 yr of age (mean 22.91 yr, SD 4.3); they were fluent English speakers and did not have a history of neurological or psychiatric illness or drug abuse. This study was approved by the Local Research Ethics Committee of the Cambridgeshire Health Authority. After description of the study to the participants, written informed consent was obtained.

#### Behavioral task.

**Fig. 1.**
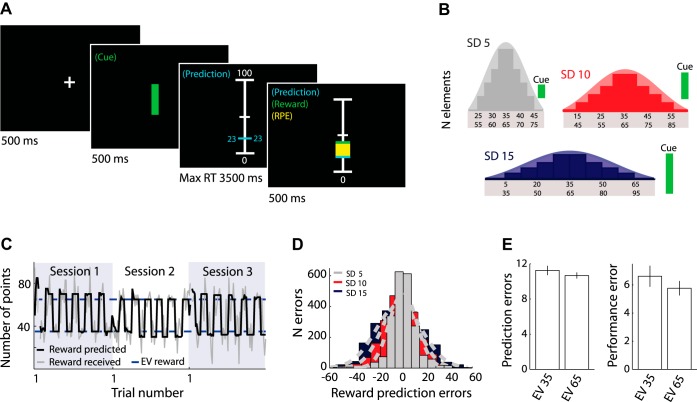
*A*: example trial of the main task. After fixation cross presentation, a small, medium, or large green bar cue signaled the (relative) fluctuation in reward value of the current distribution. After cue presentation, participants were required to indicate their prediction of the upcoming reward, after which the actual reward on that trial was shown. RT, reaction time; RPE, reward prediction error. *B*: reward distributions and cues indicating the degree of reward variability (cue: small, medium, or large green bar). Numbers listed under the distribution indicate the range of numbers per distribution: *top*, expected value (EV) 35; *bottom*, EV 65. *C*: 3 example sessions of the main task for a typical participant. In each of the 3 sessions, participants alternatingly predicted rewards drawn from 2 different distributions in small blocks of 5–8 trials, as indicated by bar cues. All participants experienced all 6 reward distributions. The order and combination of reward distributions were counterbalanced over participants. The 2 distributions in a session always had a different standard deviation (SD) and EV. *D*: number (*N*) of reward prediction errors aggregated over participants and trials. Reward prediction errors increased with SD, indicating that the experimental manipulation was successful. *E*: average (±SE) prediction errors (*left*) and performance errors (*right*) decreased for reward distributions with a higher EV, thus suggesting that participants perceived the drawn numbers as actual rewards.

The experimental task required participants to predict the magnitude of upcoming rewards as closely as possible from the past reward history. Rewards were points (i.e., numbers) drawn from six different pseudo-Gaussian distributions (SD 5, 10, or 15 and EV 35 or 65). Each trial started with a fixation cross presented on a computer monitor in front of the participants (Fig. 1*A*). After 500 ms of fixation cross presentation, a small, medium, or large green bar cue signaled the SD (5, 10, or 15) of the reward distribution from which the upcoming reward would be drawn (500 ms). Bar height was proportional to SD but did not correspond to the actual SD or to the range of the distributions. As such, the bar cue informed participants whether rewards were drawn from a distribution with a small (SD 5), medium (SD 10), or large (SD 15) level of variability without revealing the actual size of the SD and/or range. Thus these explicit cues facilitated rapid adaptation to reward variability. Importantly, the cues did not contain information on the EV of the distributions. After cue presentation, participants moved a horizontal bar with the numeric value displayed on both sides on a vertical scale (0–100) with a trackball mouse and indicated their prediction by a mouse click (within 3,500 ms). After a short delay (300 ms), the display showed the magnitude of the drawn reward as a green line and numbers on the same scale, as well as the reward prediction error on that trial (a yellow bar spanning the distance between the predicted and the received reward). Reward prediction error was conventionally defined as δ = reward received − reward predicted. Failure to make a timely prediction resulted in omission of the reward.

Each participant completed three sessions of 10 min each of the task. In each session participants alternatingly predicted from one of two conditions (i.e., distributions; Fig. 1*B*). Each condition had a run length of 42 trials, resulting in 84 trials per session. There were exactly 42 rewards per condition, ensuring that each participant received the same rewards. The two conditions in a session alternated in short blocks of five to eight trials (12 short blocks per session; 6 short blocks for each of the 2 conditions in a session). See Fig. 1*C* for an example participant. Importantly, participants could use all 42 trials to estimate the SD and EV of a condition, independent of the short block in which a trial occurred. All analyses and model fits were conducted on the 42 trials of each condition, as if these trials had been presented in direct succession. The two reward conditions (i.e., distributions) in a session never had the same SD and/or EV, and each distribution occurred only once per participant. There were six possible pairs of distributions, of which each participant saw three pairs (i.e., 1 pair per session). Sixteen participants were presented with the first combination of pairs (SD 5 EV 35 and SD 10 EV 65, SD 10 EV 35 and SD 15 EV 65, SD 15 EV 35 and SD 5 EV 65), whereas the remaining fifteen participants performed the second combination (SD 5 EV 35 and SD 15 EV 65, SD 10 EV 35 and SD 5 EV 65, SD 15 EV 35 and SD 10 EV 65). The six possible orders of the three pairs (over sessions) were counterbalanced so that each order was performed by five participants, except for the sixth order, which was performed by six participants. The order of the two distributions within a session was randomized. The order of rewards within a condition (i.e., 1 of the 6 distributions) was pseudorandomized. First, we randomized the rewards within a condition. Subsequently, we ensured that outliers did not occur in succeeding trials.

All distributions had zero skewness, no tails, and insignificant deviation from normality (Shapiro-Wilk; *P* = 0.54, 0.89, and 0.92 for SDs of 5, 10, and 15 points, respectively). However, they were slightly less “peaked” than a true Gaussian distribution, as indicated by a kurtosis of 2.6 (SD 5), 2.6 (SD 10), and 2.57 (SD 15). Initial inspection of reward prediction error data revealed that these errors increased with SD, thus indicating that the experimental manipulation was successful (Fig. 1*D*).

#### Instructions.

Participants were instructed on the experiment with the aid of a standardized MATLAB tutorial that fully informed them about the structure of the task. That is, we indicated that rewards were drawn from “pots” (i.e., distributions) with a low, medium, or large degree of variability as indicated by the bar cues. Furthermore, we specified that each of the three task sessions required participants to alternatingly predict from one of two pots (distributions), resulting in a total of six different pots (small variability *n* = 2, medium variability *n* = 2, and large variability *n* = 2). We indicated that two pots with the same degree of variability (e.g., small) would be centered at a different physical location on the scale (i.e., had a different EV). Participants were only ignorant about the exact parameter values (i.e., the EVs, SDs, and range used as well as the frequency of alternation between the 2 distributions within a session). Furthermore, although we indicated that the two distributions within a session had a different SD, we did not reveal that the two pots within a session would also have a different EV. Nor did we specify that each pot had only one of two EVs. Debriefing after the experiment revealed that participants believed that each of the six distributions had a different EV. We informed the participants that the goal of the experiment was to predict the next reward as closely as possible from the past reward history.

#### Payoff.

Participants were informed that the experiment comprised two different trial types, “main” and “control” trials, and that the gains from one main and one control trial were selected pseudorandomly and paid out to the participants at the end of the experiment. We explicitly stated that in the main trials the payoff was a fraction (10%) of the reward drawn by the computer (80% of all trials; e.g., £5 if a participant received 50 points) and that in these trials rewards were shown in green. Although participants were informed that most trials were main trials, we did not reveal the actual contingencies. This design motivated the participants to consider the drawn numbers as actual rewards. Initial inspection revealed that participants' accuracy in predicting upcoming rewards increased for distributions with higher EVs as reflected in lower prediction errors [*T*(30) = 2.27, *P* = 0.0306; Fig. 1*E*, *left*]. In addition, participants' accuracy in predicting the mean of reward distributions increased for higher EVs as reflected in lower performance errors [|predictions − EV|; *T*(30) = 2.49, *P* = 0.0186; Fig. 1*E*, *right*], thus suggesting that participants perceived the drawn numbers as rewards. To ensure that participants revealed their true predictions in an incentive-compatible way, we pseudorandomly interspersed unannounced control trials (20% of all trials). Participants were told that in these trials payoff depended on their performance, i.e., how close their prediction was to the EV of the reward distribution. Predictions that differed no more than 1 SD (in points) from the EV were rewarded with £7.50, predictions that differed more than 1 SD but less than 2 SDs from the EV led to a reward of £5, and all other predictions led to a reward of £2.50. As in the main trials, the monitor displayed the number drawn by the computer after the participant had indicated his/her prediction and did not indicate performance. However, the number drawn by the computer was shown in red to indicate the participant's “supervision.” Just as the green number, this number was a reward drawn by the computer and did not tell participants how well they were performing on that trial. Importantly, there was no indication about the control trial at the time the participants stated their prediction. Because of their unannounced occurrence, these control trials thus encouraged the participants to optimize their performance during all trials. The tutorial informed participants that they should try to predict as well as possible on every trial as they did not know at the moment of prediction whether their payoff on that trial depended on their performance or on the number drawn by the computer.

#### Practice sessions.

Prior to the main task, each participant completed two practice sessions. Here, rewards were drawn from distributions with a different SD (i.e., 7 and 14 points) and EV (i.e., 30 and 60 points). As in the main task, the height of bar cues was proportional to, but did not reflect, the actual SD or range of distributions. To familiarize participants with the trackball mouse, each participant also completed a short motor task. In each trial (total of 90 trials) participants received 3,500 ms to scroll to a number on the scale that was printed in green on top of the scale. All stimulus presentation, data acquisition, and data analyses were programmed with MATLAB and Cogent 2000 (http://www.vislab.ucl.ac.uk/cogent_2000.php).

#### Constant vs. dynamic learning rates.

Prior to investigating adaptation to reward variability, we determined whether predictions were updated with constant or dynamic learning rates. As predictions can more rapidly become stable through decreasing learning rates, we hypothesized that a reinforcement learning model with a dynamic learning rate would better fit the data. That is, in variable contexts, predictions based on constant learning rates can only become stable with low learning rates. This results in slow learning and impedes overall performance. We fit a constant learning rate Rescorla-Wagner model (Rescorla and Wagner 1972) and a dynamic learning rate Pearce-Hall model (LePelley and McLaren 2004; Li et al. 2011; Pearce and Hall 1980) to participants' prediction sequences. Both models updated predictions as a function of the reward prediction error (δ) and the learning rate (α). Learning rates were conventionally constrained to the interval [0 1].

On each successive trial *t* of the Rescorla-Wagner model, the prediction (*P*) was updated according to the prediction error (δ) multiplied by the learning rate (α):

(1) $$P_{t+1}=P_{t}+\alpha \times \delta_{t}$$

The predictions (*P*_*t*_) were initialized to the first prediction (*P*_1_) of the participant for each condition, and the constant learning rate (α) was estimated for each participant. First, we estimated the Rescorla-Wagner model using one learning rate across SD conditions. Subsequently, we adjusted this model to allow for learning rates that differed with SD, thus adding two additional free parameters.

According to Pearce-Hall, decreases in absolute prediction error across trials may be used to guide changes in learning rate (Pearce and Hall 1980). Here the dynamic learning rate (α_*t*_) depends on the weighted (η) unsigned prediction error (normalized to a value in the range [0 1]) across the past trials. The weighing factor (η; range [0 1]) regulates the extent of the gradual change in learning rate:

(2) $$\begin{array}{l} P_{t+1}=P_{t}+\alpha \times \delta_{t}\\ a_{t+1}=\eta \times \left|\frac{\delta_{t}}{100\;\text{points}}\right|+\left(1-\eta \right)\times \alpha_{t} \end{array}$$

Here large prediction errors will result in an increase in learning rate on the next trial, whereas learning rates will decrease with smaller prediction errors. When η > 0 the new learning rate depends on the previous learning rate and the previous absolute prediction error. Importantly, when η = 0 the Pearce-Hall model is equivalent to a constant learning rate Rescorla-Wagner model. Thus the Rescorla-Wagner model is nested in the more complicated Pearce-Hall via a parametric restriction. The initial learning rate (α_1_) and decay parameter (η) are the free parameters that are estimated via model fitting. First, this model was estimated using one initial learning rate for all SD conditions. Subsequently, we adjusted this model to allow for initial learning rates that differed with SD, thus adding two additional free parameters.

#### Simulated data.

We conducted a simple simulation to determine the theoretical effect of *1*) (initial) learning rate, *2*) learning rate decay, and *3*) reward variability on performance in our task. We constructed reward distributions with 20 different SDs (i.e., SD 1–SD 20). Each distribution had an EV of 0. With MATLAB, 50 reward distributions of 42 trials each (i.e., equivalent to our task) were generated for each SD by drawing random round numbers from a Gaussian distribution. Subsequently, we inspected overall performance error (|performance − EV| averaged over all trials) for learning rates between 0 and 1 (in steps of 0.01) and gradual decays in learning rate between 0 and 1 (in steps of 0.1). For each SD, learning rate, and decay, performance error was averaged over the 50 different distributions generated for each SD. The first prediction (i.e., start point) in our simulation was randomly drawn from a distribution with an EV of 15 and an SD of 2. This was motivated by the observation that participants in our task tended to predict rewards of ±50 points (50.77 ± 2.23) during the first trial of each distribution, i.e., at the middle of the scale, thus resulting in performance errors of ±15 (i.e., |50 − 35| or |50 − 65|). We removed the first prediction prior to the calculation of simulated performance error data.

**Fig. 2.**
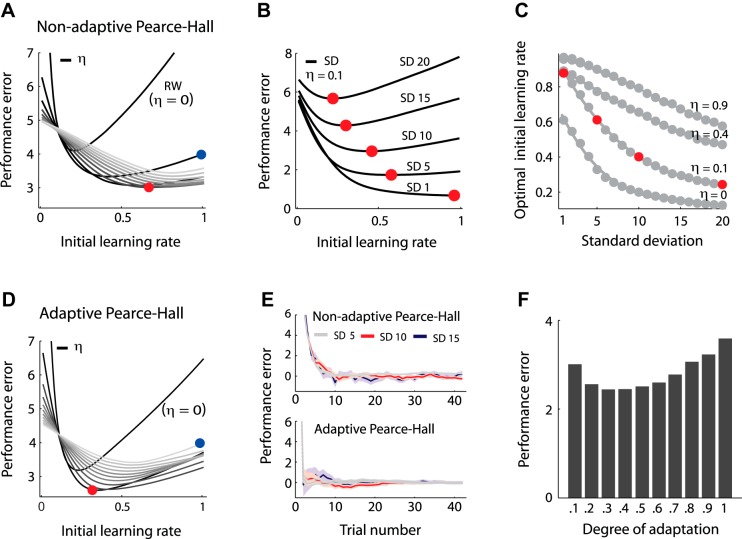
*A*: simulated overall performance error (|performance − EV| averaged over all trials) for the Pearce-Hall model (see text for details on the simulation). Each line represents performance error across different learning rates for a specific decay in learning rate (*y*-axis). Grayscale lines represent different gradual decays in learning rate (η; 0–1, in steps of 0.1). Lighter grays indicate increases in learning rate decay. When the decay in learning rate is 0, the Pearce-Hall (PH) model is equivalent to the Rescorla-Wagner (RW) model. Performance error depends on both the initial learning rate (*x*-axis) and the gradual decay in learning rate. For most (initial) learning rates performance error is lower when combined with a decaying rather than a constant learning rate. *B*: optimal initial learning rates for SDs of 1, 5, 10, 15, and 20 and a decay of 0.1. Optimal initial learning rate was quantified as the initial learning rate for which best overall performance could be achieved. The optimal initial learning rate decreases when SD increases. Each black line indicates performance error across different learning rates and represents a specific SD. Red dots indicate the optimal learning rate for each SD. *C*: optimal learning rates (gray dots and line) for different SDs (SD 1–SD 20) and multiple decays in learning rate (0, 0.1, 0.4, and 0.9). Optimal learning rates decrease when SD increases for each level of decay. Red dots correspond with the red dots in *B*, i.e., the optimal initial learning rates for SD 5, 10, 15, and 20 with a decay of 0.1. *D*: simulated overall performance error for the adaptive Pearce-Hall model where prediction errors are scaled relative to the logarithm of SD (*Eq. 4*; ν = 0.5). Grayscale lines represent different gradual decays in learning rate (η; 0–1, in steps of 0.1). Lighter grays indicate increases in learning rate decay. Although the minimum performance error is lower in the adaptive compared with the nonadaptive Pearce-Hall model (compare red dots in *A* and *D*), performance also critically depends on the initial learning rate and the gradual decrease in learning rate (compare blue dots in *A* and *D*). Thus performance may, but does not necessarily, improve with adaptation. *E*: simulated predictions with the nonadaptive (*top*) and adaptive Pearce-Hall model (*bottom*) for distributions with an SD of 5, 10, and 15, an initial learning rate of 0.5, and a gradual decay in learning rate of 0.1. Lines represent average of 200 simulated sessions. Shaded areas indicate SE. Adaptation facilitates faster learning and more similar performance error across SD conditions. *F*: relation between the degree of adaptation (*Eq. 4*; prediction errors scaled with the logarithm of SD) and performance error. Whereas scaling of prediction errors relative to but smaller than (log)SD facilitates decreases in performance error, scaling with a magnitude close to the (log)SD may limit the power of the learning rate to update predictions, resulting in increases in performance error. Thus performance may, but does not necessarily, improve with adaptation.

In line with previously reported results, simulated data (Fig. 2*A*) show that the use of dynamically decreasing learning rates facilitates substantial decreases in overall performance error (|prediction − EV| averaged over all trials) compared with the use of a constant Rescorla-Wagner learning rate (Nassar et al. 2010).

#### Adaptation to reward variability.

Performance may be further improved through scaling of prediction errors relative to reward variability. Investigating the relationship between prediction error scaling and task performance was the main goal of this study. If such adaptation indeed benefits performance, the optimal learning rate should differ for varying degrees of reward variability in the absence of prediction error scaling. The optimal learning rate was defined as the learning rate that resulted in lowest overall performance error for a specific SD in the simulated data. Simulated results show that the lowest performance error could be achieved through the use of smaller (initial) learning rates when SD increases (see Fig. 2, *B* and *C*). This relationship was present for each level of gradual decay in learning rate (see Fig. 2*C* for multiple decays). Whereas the optimal (initial) learning rate varied with the logarithm of the SD for small decays, this relation was linear for the highest decays (Fig. 2*C*). To investigate adaptation in our experimental data, we first compared model fits for *1*) a model with one (initial) learning rate across SD conditions to *2*) a model with SD-specific (initial) learning rates. If participants would adapt to reward variability, the model with SD-specific learning rates should provide a better fit of participants' prediction sequences.

To determine how well a normative model including prediction error scaling described human behavior, we divided the reward prediction error by the SD of the received rewards. This model is similar to the Pearce-Hall model (*Eq. 2*); however, in this model the reward prediction error is divided by the SD (σ_*t*_) of the received rewards. As the relationship between optimal learning rate and SD is logarithmic for lower decays (see simulations), we multiplied the adaptation parameter with the logarithm of the observed SD in a second version of the model:

(3) $$\begin{array}{l} P_{t+1}=P_{t}+\alpha_{t}\times \frac{\delta_{t}}{\omega_{t}}\\ a_{t+1}=\eta \times \left|\frac{\delta_{t}/\left(\log\right)\left(\sigma_{t}\right)}{100\;\text{points}}\right|+\left(1-\eta \right)\times \alpha_{t} \end{array}$$

Here σ_*t*_ is the SD of rewards received on *trial 1* to *trial t*. The initial expected SD of rewards σ_1,2_ was a free parameter that was estimated separately for each SD condition, thus resulting in three free parameters. The initial learning rate (α_1_) and decay parameter (η) were additional free parameters that were estimated via model fitting.

As it is conceivable that participants scale prediction errors relative to, but with a quantity smaller than, the SD, we subsequently adjusted the adaptive model by adding a free scaling parameter (ω_*t*_) on prediction errors. To obtain the scaling parameter, a free parameter (ν) that allowed for individual variation in adaptation was multiplied with the SD (σ_*t*_). As the relationship between optimal learning rate and SD is logarithmic for lower decays, we multiplied the adaptation parameter with the logarithm of the SD (σ_*t*_) in a second version of the model:

(4) $$\begin{array}{l} P_{t+1}=P_{t}+\alpha_{t}\times \frac{\delta_{t}}{\omega_{t}}\\ a_{t+1}=\eta \times \left|\frac{\delta_{t}/\omega }{100\;\text{points}}\right|+\left(1-\eta \right)\times \alpha_{t}\\ \omega_{t}=\left(1-v\right)+v\times \left(\log\right)\left(\sigma_{t}\right) \end{array}$$

The initial expected SD of rewards σ_1,2_ was a free parameter that was estimated separately for each SD condition, thus resulting in three free parameters. The initial learning rate (α_1_), decay parameter (η), and adaptation index (ν) were additional free parameters that were estimated via model fitting. ν > 0 indicates that participants adjust the initial learning rate relative to reward variability. In contrast, when ν = 0 reward prediction errors are divided by 1, resulting in no adaptation. ν was constrained to the interval [0 1] where a value of 1 indicates adaptation to (the logarithm of) the SD. Importantly, this adaptive Pearce-Hall model can be transformed into the simpler nonadaptive Rescorla-Wagner and Pearce-Hall models by imposing a set of constraints on the parameters. Specifically, for ν = 0 this model is equivalent to the nonadaptive Pearce-Hall model (*Eq. 2*). In addition, when ν = 0 and η = 0 this model is equivalent to the Rescorla-Wagner model (*Eq. 1*).

#### Model fitting and comparison.

We estimated the free parameters of each model using a constrained search algorithm (fmincon in MATLAB) to minimize the total squared difference between participants' predictions and prediction sequences generated by the model. Models were fitted for each participant separately (i.e., using an individual set of free parameters) using all SD conditions and trials of the main task (*n* = 252; 6 distributions × 42 trials). For model comparison within participants, we used the Akaike information criterion (AIC), which penalizes the number of free parameters to determine the overall best model. For model comparisons at the group level, AIC values were aggregated over all participants for each model. Thus this approach allowed us to conduct model comparisons on the individual as well as the group level. In addition, as the Rescorla-Wagner (*Eq. 1*) and Pearce-Hall (*Eq. 2*) models are nested in the adaptive Pearce-Hall model (*Eq. 4*) via restrictions on model parameters, we used likelihood ratio tests to investigate whether superior fits of the adaptive model were better than chance level. Thus we determined whether the improvement in fit gained by allowing the adaptation parameter to be free was warranted.

As adaptation presumably required participants to learn the structure of the task and the degree of reward fluctuation associated with SD cues, it was hypothesized that prediction error scaling relative to reward variability would be reduced or absent during the practice sessions. Consequently, we also obtained the best-fitting model parameters for each participants' practice sessions (*n* trials: 168) and repeated the model comparisons.

#### Adaptation to reward variability and learning efficiency.

To test our central hypothesis, we determined whether scaling of prediction errors relative to reward variability would be related to improvements in learning in humans. Efficient learning requires individuals to rapidly acquire stable and accurate predictions in contexts with varying degrees of reward variability. Higher overall efficiency in learning should be reflected in smaller overall performance error (|prediction − EV| averaged over all trials). Consequently, *1*) overall performance error, *2*) final performance error, and *3*) final prediction (in)stability were used as the main measures of learning efficiency. Final performance error was quantified as the average performance error during the final short block of the task (±trial 36:42). Final prediction instability pertained to the SD of participants' predictions in the final short block. Importantly, scores on the different outcome measures could be highly correlated, e.g., increases in (final) performance error could result from unstable predictions rather than stable predictions distant from the EV. Indeed, high correlations (Spearman's ρ > 0.80) were present between overall performance error, final performance error, and prediction instability. Thus we used overall performance error as the representative outcome measure for learning efficiency. Failure of adaptation was hypothesized to have a larger effect on performance error magnitude for higher SDs (see Fig. 2*E*). Specifically, adapters (ν > 0) and nonadapters (ν = 0) may show similar accuracy in predicting the mean when SD is low but differ in their performance for higher SDs. Consequently, (dis)similarity in performance for different SD conditions was used as an additional measure of learning efficiency. Performance dissimilarity was quantified as the SD of overall performance error across the different SD conditions.

Although the simulated data suggest that scaling of prediction errors relative to reward variability may improve performance (compare red dots in Fig. 2*A* and Fig. 2*D* and compare Fig. 2E, *top* and *bottom*; simulated data), performance also critically depends on the gradual decay in learning rate and the initial learning rate (compare blue dots in Fig. 2*A* and Fig. 2*D*; simulated data). Thus an increase in computational demands required for adaptation may, for instance, interfere with optimal learning rate decay. In addition, scaling with a magnitude close to the (logarithm of the) SD may limit the power of the learning rate to update predictions (see Fig. 2*F*). Consequently performance may, but does not necessarily, improve with adaptation as predicted by normative models.

To allow for a nonlinear relation between learning efficiency (overall performance error and dissimilarity in performance error across SD conditions) and the degree of adaptation (ν; *Eq. 4*), we conducted quadratic regressions. The initial learning rate (α_1_; *Eq. 4*) and the gradual decrease in learning rate (η; *Eq. 4*) were used as additional independent variables in the regressions:

(5) $${\text{Y}}_{1}\left(\text{perf. error}\right)=\beta_{0}+\beta_{1}\left(v\right)+\beta_{2}\left(\eta \right)+\beta_{3}\left(\alpha_{1}\right)+\beta_{4}\left(v^{2}\right)+\beta_{5}\left(\eta^{2}\right)+\beta_{6}\left(\alpha_{1}^{2}\right)+\in$$

(6) $${\text{Y}}_{2}\left(\text{SD}\left(\text{perf. error}\;\text{across}\;\text{SDs}\right)\right)=\beta_{0}+\beta_{1}\left(v\right)+\beta_{2}\left(\eta \right)+\beta_{3}\left(\alpha_{1}\right)+\beta_{4}\left(v^{2}\right)+\beta_{5}\left(\eta^{2}\right)+\beta_{6}\left(\alpha_{1}^{2}\right)+\in$$

To obtain standardized regression coefficients, all independent and dependent variables were *z*-transformed.

## RESULTS

### 

#### Participants used dynamic learning rates.

**Fig. 3.**
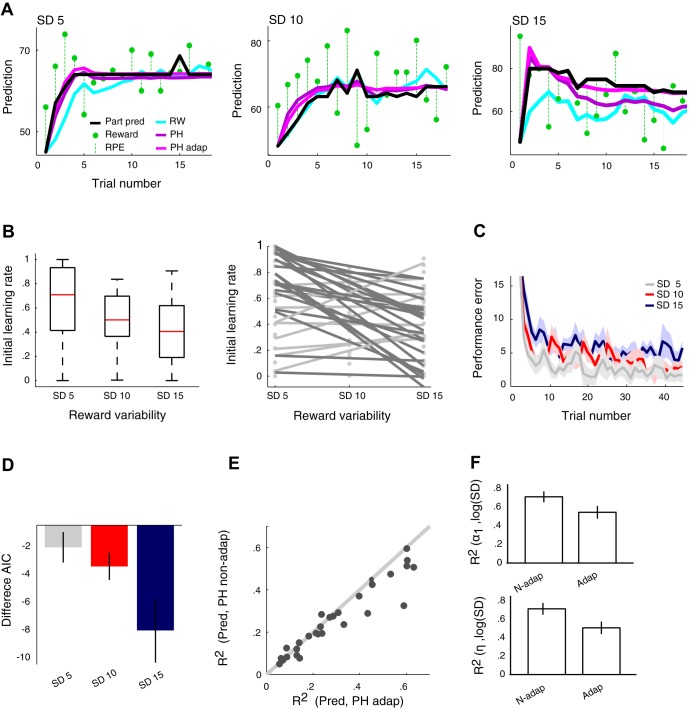
*A*: observed and modeled predictions of reward in a typical participant for the constant learning rate Rescorla-Wagner model, the nonadaptive Pearce-Hall model, and the adaptive Pearce-Hall model (*Eq. 4*; prediction errors scaled relative to the logarithm of SD). The Pearce-Hall models with dynamic learning rate provided a superior fit to participants' prediction sequences compared with the constant learning rate Rescorla-Wagner model. In addition, the adaptive Pearce-Hall model provided a better fit compared with the nonadaptive Pearce-Hall model. Whereas the difference in fit between the 2 Pearce-Hall models was relatively small for lower SDs, this difference was pronounced for the high-SD condition (*right*). *B*, *left*: median initial learning rates decreased significantly for increases in SD, suggesting adaptation to reward variability. *Right*: changes in initial learning rates as a function of SD in individual participants. Markers provide estimated initial learning rates; lines are least-squares lines fitted through these data points. Whereas the majority of participants (dark gray lines) decreased initial learnings when SD decreased, some participants used the same initial learning rate across different SDs or increased initial learning rates when SD increased (light gray lines). *C*: average (±SE) performance error (|prediction − EV|) data across all participants and trials showing that participants continued to update their predictions until the final trials of each condition. *D*: difference in Akaike information criterion (AIC) values between the adaptive and nonadaptive Pearce-Hall models increased for increases in SD, indicating that prediction error scaling becomes more important when SD increases. *E*: *R*^2^ values from linear regressions where modeled predictions from the nonadaptive (*Eq. 2*) and adaptive (*Eq. 4*) Pearce-Hall models were the independent variables and participants' predictions were the dependent variable. Most participants' predictions were better explained by the adaptive Pearce-Hall model. *F*, *top*: the logarithm of SD provides a better predictor of learning rate (average *R*^2^ ± SE) for the nonadaptive compared with the adaptive model. Importantly, for these analyses, initial learning rates and learning rate decay (and the degree of adaptation) were allowed to vary across SD conditions for the nonadaptive as well as the adaptive model. *Bottom*: the logarithm of SD provides a better predictor of learning rate decay (average *R*^2^ ± SE) for the nonadaptive compared with the adaptive model. Thus initial learning rates and learning rate decays were more similar across SD conditions after adaptation. Part pred, participants' predictions; n-adap, nonadaptive; adap, adaptive.

As dynamic learning rates can improve learning in variable contexts, we inspected whether participants decelerated learning across trials. Model comparisons showed that the Pearce-Hall model with a dynamic learning rate (*Eq. 2*) provided a superior fit to participants' prediction sequences compared with a constant learning rate Rescorla-Wagner model (see Table 1 for model comparisons; see Fig. 3*A* for a typical participant). Inspection of individual model fits revealed that the Rescorla-Wagner model performed best in only a small minority of the participants (3/31). This result validates the nesting of adaptation to reward variability in a Pearce-Hall model.

**Table 1.** Quality of model fits to participants' prediction sequences using a separate set of parameters for each participant

Model	RW	PH	PH—SD-Specific α_1_	Linear Adaptive PH: ν = 1	Log Adaptive PH: ν = 1	Linear Adaptive PH: ν = [0 1]
PH	dAIC: −439.67					
	$\chi_{31}^{2}$ = 501.67, *P* < 0.001					
PH—SD-specific α_1_	dAIC: −505.78	dAIC: −66.10				
	$\chi_{93}^{2}$ = 691.78, *P* < 0.001	$\chi_{62}^{2}$ = 190.00, *P* < 0.001				
Linear adaptive PH	dAIC = −544.18	dAIC = −104.51	dAIC = −38.41			
Fixed parameter adaptation: ν = 1	$\chi_{124}^{2}$ = 965.18, *P* < 0.001	$\chi_{93}^{2}$ = 463.50, *P* < 0.001	$\chi_{31}^{2}$ = 273.40, *P* < 0.001			
Log adaptive PH	dAIC: −621.05	dAIC: −181.38	dAIC: −115.278	dAIC: −76.87		
Fixed parameter adaptation: ν = 1	$\chi_{124}^{2}$ = 869.29, *P* < 0.001	$\chi_{93}^{2}$ = 367.62, *P* < 0.001	$\chi_{31}^{2}$ = 177.51, *P* < 0.001			
Linear adaptive PH	dAIC: −635.86	dAIC: −196.18	dAIC: −130.08	dAIC: −91.67	dAIC: −14.80	
Free adaptation parameter: ν = [0 1]	$\chi_{155}^{2}$ = 945.86, *P* < 0.001	$\chi_{124}^{2}$ = 444.18, *P* < 0.001	$\chi_{62}^{2}$ = 254.08, *P* < 0.001	$\chi_{31}^{2}$ = 149.79, *P* < 0.001	$\chi_{31}^{2}$ = 76.57, *P* < 0.001	
Log adaptive PH	dAIC: −671.02	dAIC: −231.34	dAIC: −165.24	dAIC: −35.16	dAIC: −35.16	dAIC: −35.16
Free adaptation parameter: ν = [0 1]	$\chi_{155}^{2}$ = 981.02, *P* < 0.001	$\chi_{124}^{2}$ = 479.34, *P* < 0.001	$\chi_{62}^{2}$ = 289.24, *P* < 0.001	$\chi_{31}^{2}$ = 184.94, p < 0.001	$\chi_{31}^{2}$ = 111.73, *P* < 0.001	

RW, Rescorla-Wagner; PH, Pearce-Hall; SD, standard deviation; α_1_, initial learning rate; ν, adaptation to reward variability; dAIC, difference in Akaike information criterion value.

#### Adaptation to reward variability.

To investigate adaptation to reward variability, we first determined whether model fits for the Pearce-Hall model improved by including SD-specific initial learning rates. Indeed, model fits improved when initial learning rates could differ across SD conditions (Table 1). Initial learning rates decreased significantly for increases in SD [repeated-measures ANOVA: *F*(2,60) = 11.0788, *P* = 8.0374e-005, all 1-tailed post hoc tests: *P* < 0.0167 (value required for Bonferroni correction); Fig. 3*B*, *left*]. Whereas this effect was present in the majority of participants, some participants (9/31) used similar or increasing initial learning rates when SD increased (Fig. 3*B*, *right*). Importantly, the superior fit of a model with SD-specific learning rates did not solely result from the first few trials, as model fits computed after exclusion of the initial 10 trials of each distribution also resulted in superior performance of the model with SD-specific learning rates (difference in AIC participant-specific model parameters = −152.23; χ_124_^2^ = 276.23, *P* < 0.001; performance error data across trials show that participants still updated their predictions after the first 10 trials; Fig. 3*C*).

As each session included two conditions that alternated in short blocks, initial learning rates for the first condition potentially depended on the second condition in that session. Specifically, initial learning rates for SD 10 conditions might increase if the second condition in a session has a higher SD (i.e., SD 15). However, initial learning rates estimated separately for the two SD 10 conditions did not differ significantly when paired with SD 5 compared with SD 15 [*T*(60) = 0.7424, *P* = 0.4607]. This finding renders the presence of additional contextual effects on adaptation unlikely.

To facilitate formal tests of adaptation we adjusted the Pearce-Hall model to include prediction error scaling to reward variability (*Eqs. 3* and *4*). Participants' predictions were better fit by adaptive Pearce-Hall models that scaled prediction errors with (*Eq. 3*) or relative to (*Eq. 4*) the (log)SD, compared with the nonadaptive Pearce-Hall model (Table 1). Even though the limited number of trials posed a restriction on statistical power on the individual level, the adaptive Pearce-Hall models provided a significantly better fit in the majority of participants (16/31) compared with the simpler models, evidenced by lower AIC values and significant likelihood ratio tests (see Fig. 3*A* for a typical participant). The minority of participants for whom the likelihood ratio tests were not significant comprised both individuals (9/31) in whom initial learning rates did not decrease for increases in SD (see above) as well as individuals (6/31) in whom adaptation occurred but failed to reach significance, presumably because of the limited number of trials. In line with the notion of individual differences in the degree of adaptation, Pearce-Hall models that included a free parameter for adaptation (*Eq. 4*) outperformed Pearce-Hall models that used a fixed adaptation parameter (*Eq. 3*; Table 1). Of the two adaptive models with a free adaptation parameter, the logarithmic adaptive model provided a slightly better fit to participants' prediction sequences compared with the linear adaptive model (Table 1). Consequently, this model was used for subsequent analyses. The difference in fit between the nonadaptive and adaptive Pearce-Hall models was most pronounced for high-SD conditions [*F*(2,60) = 4.16, *P* = 0.0203; Fig. 3*D*; also compare Fig. 3*A*, *left* and *right*]. Modeled predictions from the log adaptive Pearce-Hall model (*Eq. 4*) better predicted participants' predictions compared with modeled predictions from the nonadaptive Pearce-Hall model (*Eq. 2*; Wilcoxon signed-rank test on linear regression coefficients: *Z* = −3.0571, *P* = 0.0022; Fig. 3*E*). In line with this finding, estimated adaptation parameters differed significantly from zero [0.5133 ± 0.3495; *T*(1,30) = 8.1757, *P* < 0.001]. Although all model fits were superior for distributions with a higher EV (i.e., EV 65 vs. EV 35), the (log)adaptive Pearce-Hall model provided a better fit to participants' prediction sequences compared with the nonadaptive Pearce-Hall model for both EVs (difference in AIC = −110.93 and −269.46; χ_124_^2^ = 358.93, *P* < 0.001 and χ_124_^2^ = 517.47, *P* < 0.001 for EV 35 and EV 65, respectively). Superior performance of this adaptive model compared with the nonadaptive Pearce-Hall model was confirmed by pooling prediction sequence data across participants and fitting both models on the aggregated data using one set of free parameters across participants (difference in AIC = −3.09; χ_4_^2^ = 11.09, *P* = 0.0256). Finally, we compared the log adaptive Pearce-Hall model with a free adaptation parameter (*Eq. 4*) to the nonadaptive Pearce-Hall (*Eq. 2*) model using the more conservative Bayesian information criterion (BIC). Compared with AIC, BIC has a greater preference for simplicity and penalizes models with more parameters more heavily (Lewandowski and Farrell 2011). With BIC, this adaptive Pearce-Hall model initially provided an inferior fit to participants' behavior compared with the nonadaptive Pearce-Hall model (difference in BIC = 203.2948). However, this negative effect resulted from punishment for the three free parameters used to estimate the initial expected SD of rewards, not from the penalty for the adaptation parameter. After the requirement to estimate the initial expected SD of rewards was removed, the adaptive model outperformed the nonadaptive model (across participants difference in BIC = −58.56; on aggregated data difference in BIC = −1.38). Specifically, we fit the adaptive and nonadaptive models to participants' prediction sequences from the third trial onward, using the SD of rewards received over the first two trials as the initial SD of rewards. On subsequent trials the SD of rewards was updated as specified in *Eq. 4*. In summary, these results suggest that participants scale prediction errors in addition to decreasing the learning rate across subsequent trials.

Adaptation to reward variability only became apparent during the main task as participants' predictions in the two practice sessions were better fit by the nonadaptive Pearce-Hall model (difference in AIC across participants = −134.51; χ_124_^2^ = 382.51, *P* = 1). This result suggests that adaptation required participants to learn the structure of the task and the degree of reward variability associated with the SD cues.

The presence of adaptation to reward variability implies that dynamic learning rates varied with scaled prediction errors. To describe this effect, we fitted participants' prediction sequences for each SD condition separately using the (logarithmic) adaptive Pearce-Hall model. Thus initial learning rates, learning rate decay, and the degree of adaptation were allowed to vary across SD conditions. As expected, simple linear regressions showed that (log)SD was a significantly better predictor of initial learning rate for the nonadaptive Pearce-Hall model compared with the adaptive Pearce-Hall model (Wilcoxon signed-rank test on *R*^2^: *Z* = −2.2732, *P* = 0.0203; Fig. 3*F*, *top*). In addition, learning rate decay was better predicted by (log)SD for the nonadaptive compared with the adaptive model (Wilcoxon signed-rank test on *R*^2^: *Z* = −2.3516, *P* = 0.0187; Fig. 3*F*, *bottom*). Thus, as expected from improved model fits for the adaptive model, initial learning rates and learning rate decays were more similar across SD conditions after adaptation.

#### Adaptation and learning efficiency.

Importantly, adaptation to reward variability may serve to make learning resistant to fluctuations in reward value. Although scaling of prediction errors relative to reward variability should benefit performance, scaling with the SD may limit the power of the learning rate to update predictions (Fig. 2*F*). Thus we tested for a quadratic relationship between the degree of adaptation (ν; *Eq. 4*) and overall performance. As performance also critically depends on the gradual decay in learning rate and the initial learning rate, these parameters were used as additional regressors.

**Fig. 4.**
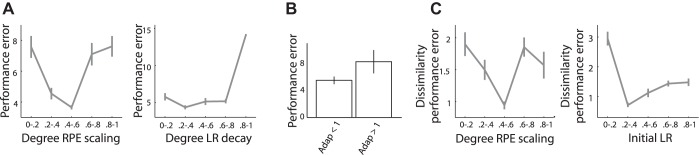
*A*, *left*: overall performance error (|prediction − EV| averaged over all trials) varied significantly with the estimated degree of prediction error scaling. Whereas performance error decreased for adaptation indexes up to ν = 0.4–0.6 (i.e., approximately half the logarithm of the SD), higher adaptation indexes were associated with increases in performance error. *Right*: relationship between learning rate (LR) decay and performance error. Performance errors slightly decreased for small increases in learning rate decay (η) but increased substantially for larger decays (>0.6–0.8). Adaptation indexes and learning rate decays were divided into 5 bins of equal width. Subsequently, performance errors were averaged over all adaptation/learning rate decay indexes in a certain bin. *B*: increases in performance error in those individuals who scaled prediction error with a quantity greater than the log SD. *C*, *left*: dissimilarity in performance error across SD conditions was lower for individuals who scaled prediction errors to a value up to ν = 0.4–0.6 (i.e., approximately half the SD) but not for those who adapted with larger values. *Right*: relationship between initial learning rate and performance error. Performance error was more similar for initial learning rates (α_1_) of ∼0.2–0.4 but became more dissimilar with smaller and larger learning rates. Adaptation indexes and initial learning rates were divided into 5 bins of equal width. Subsequently, similarity in performance error was averaged over all adaptation indexes/initial learning rates in a certain bin.

We observed a significant quadratic relationship between the individual degree of prediction error scaling and overall performance error (*P* = 0.0067; Table 2; Fig. 4*A*, *left*). Whereas performance error decreased for adaptation indexes up to ν = ∼0.5 (i.e., half the logarithm of the SD), higher adaptation indexes were associated with increases in performance error (Fig. 4*A*, *left*). Analyses using the extent of SD-dependent changes in learning rate (Fig. 3*B*, *right*) as an alternative measure for adaptation confirmed this result [β_1_^2^ = 0.1614, *T*(24) = 3.1066, *P* = 0.0048]. These results imply that efficient adaptation required scaling of prediction errors relative to, but smaller than, the (log)SD, in line with the simulated data (Fig. 2*F*). The tight relationship between the simulated and experimental data suggests that participants tended to scale their prediction errors in an optimal manner. This relationship furthermore implies that the estimated adaptation parameters provided a good fit of participants' behavior, i.e., unreliable fits might have resulted in erroneous adaptation parameters unlikely to correlate with (raw) performance error data. To further investigate the extent of prediction error scaling in relation to performance, we repeated model estimation for the log adaptive model without any constraints on the adaptation parameter. Seven of the 31 participants scaled prediction errors with a quantity larger than the log SD. These participants presented with significantly larger performance errors compared with individuals who scaled prediction errors with a quantity smaller than the SD [*T*(29) = 1.9937, *P* = 0.0278; Fig. 4*B*]. This result shows how participants can make errors and deviate from theoretical predictions.

**Table 2.** Parameter estimates and statistics for quadratic regressions predicting overall performance error

					95% Confidence Interval	
	β	SE	*t*(24)	*P*	Lower bound	Upper bound
ν	0.063	0.107	0.594	0.558	−0.157	0.283
η	0.056	0.167	0.337	0.739	−0.289	0.401
α_1_	0.078	0.172	0.454	0.654	−0.277	0.434
ν^2^	0.388	0.131	2.970	0.007	0.118	0.658
η^2^	0.537	0.121	4.427	0.000	0.287	0.788
$\alpha_{1}^{2}$	0.053	0.129	0.409	0.686	−0.214	0.320
Intercept	−0.947	0.229	−4.126	0.000	−1.420	−0.473

Fitted model: *F*(6,24) = 12.521, *P* = 0.000, *R*^2^ adjusted = 0.697. SE, standard error; ν, adaptation to reward variability; η, gradual decay in learning rate; α, initial learning rate.

Performance error not only varied with adaptation but also depended on the gradual decay in learning rate (*P* = 0.0002; Table 2; Fig. 4*A*, *right*). Performance errors slightly decreased for small increases in learning rate decay but increased extensively for larger decays (Fig. 4*A*, *right*). Thus whereas gradual deceleration of learning benefits performance, rapid deceleration results in preliminary completion of learning.

Regressions conducted for each SD separately showed that whereas the quadratic parameter for adaptation had a significant effect on performance error for higher SDs, there was only a trend-level effect when SD was 5 (*P* = 0.079, 0.013, and 0.011 for SD 5, SD 10, and SD 15, respectively; Tables 3–5). Gradual decay in learning rate significantly impacted on performance error for each of the SDs separately (*P* = 0.007, 0.000, and 0.0004 for SD 5, SD 10, and SD 15 respectively; Tables 3–5). Although learning rate decay had a larger effect on performance in the small-SD condition, compared with prediction error scaling both forms of adaptation had a similar contribution to performance in the large-SD condition (see Table 2). Thus learning rate decay and prediction error scaling can be considered separate, additional processes that impact on performance. Whereas learning rate decay benefits performance independent of SD magnitude, the effect of prediction error scaling on performance increases when SD increases.

**Table 3.** Parameter estimates and statistics for quadratic regressions predicting performance error for SD 5

					95% Confidence Interval	
	β	SE	*t*(24)	*P*	Lower bound	Upper bound
ν	0.100	0.129	0.778	0.444	−0.166	0.367
η	0.212	0.202	1.050	0.304	−0.205	0.630
α_1_	−0.084	0.209	−0.402	0.691	−0.514	0.347
ν^2^	0.291	0.158	1.837	0.079	−0.036	0.617
η^2^	0.437	0.147	2.973	0.007	0.134	0.740
$\alpha_{1}^{2}$	−0.009	0.157	−0.060	0.952	−0.333	0.314
Intercept	−0.695	0.278	−2.501	0.020	−1.268	−0.121

Fitted model: *F*(6,24) = 7.269, *P* = 0.000, *R*^2^ adjusted = 0.556.

**Table 4.** Parameter estimates and statistics for quadratic regressions predicting performance error for SD 10

					95% Confidence Interval	
	β	SE	*t*(24)	*P*	Lower bound	Upper bound
ν	0.142	0.114	1.254	0.222	−0.092	0.377
η	−0.058	0.178	−0.327	0.746	−0.426	0.309
α_1_	0.213	0.184	1.160	0.257	−0.166	0.592
ν^2^	0.372	0.139	2.667	0.013	0.084	0.659
η^2^	0.605	0.129	4.675	0.000	0.338	0.871
$\alpha_{1}^{2}$	−0.081	0.138	−0.588	0.562	−0.366	0.203
Intercept	−0.866	0.245	−3.542	0.002	−1.371	−0.362

Fitted model: *F*(6,24) = 10.545, *P* = 0.000, *R*^2^ adjusted = 0.656.

**Table 5.** Parameter estimates and statistics for quadratic regressions predicting performance error for SD 15

					95% Confidence Interval	
	β	SE	*t*(24)	*P*	Lower bound	Upper bound
ν	−0.041	0.124	−0.330	0.744	−0.298	0.216
η	0.022	0.195	0.111	0.913	−0.381	0.424
α_1_	0.084	0.201	0.416	0.681	−0.331	0.499
ν^2^	0.419	0.153	2.748	0.011	0.104	0.734
η^2^	0.451	0.142	3.182	0.004	0.158	0.743
$\alpha_{1}^{2}$	0.197	0.151	1.307	0.204	−0.114	0.509
Intercept	−1.033	0.268	−3.857	0.001	−1.586	−0.480

Fitted model: *F*(6,24) = 8.121, *P* = 0.000, *R*^2^ adjusted = 0.587.

Scaling of prediction errors relative to SD should not only facilitate improved overall performance but also result in similar learning across different levels of reward fluctuation. Indeed, dissimilarity in performance error (quantified as the standard deviation in performance error across SD conditions) was lower for individuals who adapted to a value up to ν = ∼0.5 [i.e., half the logarithm of the (log)SD] but not for those who adapted with larger values (*P* = 0.0253; Table 6; Fig. 4*C*, *left*). Similarity in performance error across SD conditions also depended on the initial learning rate (*P* = 0.0006; Table 6; Fig. 4*C*, *right*). Performance error across SD conditions was more similar for learning rates of ∼0.2–0.4 but became somewhat more dissimilar for larger learning rates and much more dissimilar with smaller initial learning rates (Fig. 4*C*, *right*). These results show that optimal adaptation is related to improved performance in variable contexts.

**Table 6.** Parameter estimates and statistics for quadratic regressions predicting dissimilarity in performance error across SD conditions

					95% Confidence Interval	
	β	SE	*t*(24)	*P*	Lower bound	Upper bound
ν	−0.218	0.146	−1.495	0.148	−0.518	0.083
η	−0.339	0.228	−1.485	0.150	−0.810	0.132
α_1_	0.456	0.235	1.940	0.064	−0.029	0.942
ν^2^	0.426	0.179	2.385	0.025	0.057	0.794
η^2^	0.228	0.166	1.377	0.181	−0.114	0.570
$\alpha_{1}^{2}$	0.694	0.177	3.926	0.001	0.329	1.058
Intercept	−1.304	0.313	−4.161	0.000	−1.951	−0.657

Fitted model: *F*(6,24) = 4.858, *P* = 0.002, *R*^2^ adjusted = 0.436.

As individual variability in adaptation to reward fluctuation could be related to the acquisition of a proper estimate of the level of variability, we inspected debriefing questionnaires. These questionnaires revealed that whereas individuals with a higher degree of adaptation (ν > 0) correctly indicated which session was most difficult in terms of the level of variability, none of the participants with an adaptation index ν < 0.1 ranked the sessions correctly. This result suggests that adapters in our task seem to acquire better estimates of the variability.

## DISCUSSION

This study investigated whether human individuals achieve superior performance through scaling of prediction errors relative to reward variability. Model comparisons confirmed that participants adapted learning rates to reward variability, in addition to deceleration of learning rates across subsequent trials (Nasser et al. 2010). Improvements in individual performance, assessed as accuracy in predicting means of reward distributions, occurred for gradual decreases in learning rates and scaling of prediction errors relative to, but smaller than, the SD. Indeed, scaling of prediction errors with a quantity exceeding the (log)SD resulted in impaired performance. Importantly, performance was more similar across SD conditions for optimal adapters. These results imply that efficient adaptation makes learning more robust to changing variability.

The positive relationship between prediction error scaling and task performance implies that increased computational resources required for adaptation did not interfere with additional task requirements including use of decreasing learning rates. Specifically, the absence of learning rate decay or very steep decays in learning rate in combination with prediction error scaling can impair performance (see Fig. 2*D*). If participants had used suboptimal initial learning rates and learning rate decays when scaling prediction errors, the degree of adaptation alone might not have been a significant predictor of performance error. This observation suggests that participants behaved in a near-optimal manner in line with the simulations. However, some (7/31) participants scaled prediction errors with a quantity exceeding the (log)SD, resulting in impaired performance. Such violation from theoretical predictions stresses the importance of comparing human behavior to predictions made by normative models (Preuschoff and Bossaerts 2007).

It is readily understandable how the observed adaptation to the predictable variability of rewards is essential for learning. Whereas a reward prediction error of a particular magnitude might be very meaningful in an environment in which rewards fluctuate less, a similar-sized error is not very meaningful when rewards vary with similar magnitude. Consequently, reward prediction errors should be scaled to variability for appropriate updating of predictions. The impact of such scaling on performance error should increase as SD increases. Indeed, whereas the extent of prediction error scaling had a significant effect on performance error for SD 10 and SD 15, there was only a trend-level effect for SD 5. Importantly, this procedure would furthermore enable individuals to detect changes in the statistics of the environment, such as a change in EV and SD of a reward probability distribution. Although previous studies showed that participants can successfully detect changes in distributions (Berniker et al. 2010; Nassar et al. 2010; Payzan-LeNestour and Bossaerts 2011), they did not identify an optimal degree of prediction error scaling or investigate the relation of such adaptation to task performance, which was the topic of the present study. Furthermore, none of these studies reported adaptation to reward variability in a stable, i.e., nonvolatile, environment.

A positive relation between learning rate adaptation, learning, and performance is implicit in Bayesian models of optimal learning (Jaynes 1986). This theorem specifies that each source of information should be weighted according to its reliability (or conversely, uncertainty). Surprising outcomes such as a large prediction error in a distribution with low variability should lead to larger updates in predictions, as they render previous predictions less reliable. As such, adaptation to variability may lead to optimal performance as predicted by Bayesian models of learning. Although Bayesian studies on learning did not correlate the individual degree of adaptation to performance, they did show that human individuals behave in an optimal or near-optimal manner as predicted by Bayesian decision theory in a number of tasks varying from sensorimotor learning to perceptual decision-making (Kording and Wolpert 2004; O'Reilly et al. 2012; Stocker and Simoncelli 2006; Yuille and Kersten 2006).

The substantial variability in the degree of adaptation as observed in the present study prompts the question of why some individuals adapt better than others. Whereas the adaptive models provided a significantly better fit in the majority of participants, some participants were fit equally well by adaptive and nonadaptive Pearce-Hall models. Individual variability is often thought to reflect differences in information-processing power (Koechlin and Hyafil 2007; O'Reilly et al. 2012), limitations of which may interfere with acquiring a proper estimation of the variability and thus hampering adaptation. Indeed, superior adapters were better at estimating the variability of each distribution, as apparent from debriefing questionnaires, in line with improvements in performance as observed on the task. In addition, adaptation only became apparent after the practice sessions. This result indicates that participants required information processing power to learn the structure of the task and the degree of reward variability associated with the SD cues in order to adapt.

In addition, in some participants none of the Pearce-Hall models (adaptive or nonadaptive) provided a good fit (Fig. 3*E*), three of whom were best fit by the constant learning rate Rescorla-Wagner model. Overall performance was lower in these participants, presumably related to a combined failure to scale prediction errors relative to reward variability and to use decreasing learning rates, potentially suggesting disengagement from the task. Importantly, omission of the participants who were best fit by the Rescorla-Wagner model did not significantly alter our findings on the relation between prediction error scaling and task performance.

Performance depended not only on the extent of prediction error scaling but also on the gradual decay in learning rate. Specifically, performance improved for gradual decays in learning rate but decreased as the decay increased. In contrast to prediction error scaling, learning rate decay impacted similarly on performance error for the different SDs. Thus learning rate decay and prediction error scaling are separate forms of adaptation that differentially impact on performance. It is crucial to behaviorally separate these two adaptation processes, as they may have different neural substrates, which future studies could examine. The observation that learning rates decayed across subsequent trials is in line with a previous study on belief-updating that required participants to predict the next number in a sequence (Nassar et al. 2010). Nassar et al. (2010) mainly focused on learning rate decreases across subsequent trials, whereas here we investigated the effect of prediction error scaling on performance. Thus we quantified the separate effects of learning rate decay and prediction error scaling prior to investigating the relation between prediction error scaling and performance. A secondary difference between the two studies is the absence of volatility (i.e., unexpected changes in outcome distributions) in our study. Volatility would confound our study goals, as participants may underestimate outcome variability under volatile conditions (Nasser et al. 2010). In volatile conditions the participant must decide which prediction errors represent “fundamental changes” in the underlying distribution and which prediction errors are the results of noise. Therefore, to isolate prediction error scaling from this “fundamental change point” detection, we performed this study in the absence of volatility. Finally, whereas Nassar et al. (2010) investigated learning about numerical (nonreward) outcomes, here we focused specifically on adaptation to reward variability. This is a crucial difference, as a wealth of studies have revealed specialized encoding of reward prediction errors in midbrain dopamine neurons and in the human ventral striatum (Garrison et al. 2013; Schultz et al. 1997). To incentivize the participants to perceive the drawn numbers as actual rewards, the payoff in our main trials (80% of all trials) depended on the reward drawn by the computer. The finding that performance predicting upcoming rewards and the EV of reward distributions increased for distributions with higher EVs suggests that this manipulation was successful. It must be noted, though, that during the incentive-compatible control trials, where the participants had to predict the EV, not the rewards, the measured prediction errors do not constitute reward prediction errors. Importantly, omission of these control trials did not significantly impact on the results. However, as the control trials were unannounced, participants presumably perceived the payoff to depend on the error estimating the EV for each trial.

The observed adaptation to reward variability involved scaling reward prediction errors relative to SD. It must be noted, though, that an alternative way for achieving the observed adaptation would be for learning rates to directly adapt to SD. Although the present study cannot distinguish between these possibilities, the scaling of prediction errors is the most effective strategy for adapting to variability according to least-squares learning theory (Preuschoff and Bossaerts 2007). Importantly, the task parameters identified in this study can be used in combination with human imaging methods to investigate this hypothesis. A neural basis for this mechanism might consist of the scaling of dopamine reward prediction error responses to SDs of reward probability distributions (Tobler et al. 2005). As such, dopamine reward prediction errors elicit the same excitatory and inhibitory neuronal responses with narrower reward distribution as larger errors do with wider distributions. Although human brain studies have not yet investigated the encoding of SD-normalized reward prediction errors, a recent study showed that striatal BOLD prediction error responses reflected reward probability but not expected reward magnitude (Park et al. 2012), which is in general agreement with the earlier dopamine study (Tobler et al. 2005). Importantly, if prediction errors are encoded in a normalized manner, learning rates should be encoded in an absolute manner, i.e., unscaled by SD. Indeed, previous studies reported that BOLD responses in the paracingulate and anterior cingulate cortex, the cuneus, and the prefrontal cortex reflect variations in absolute learning rate (Behrens et al. 2007; Krugel et al. 2009; Payzan-LeNestour et al. 2013; Vilares et al. 2012). Although additional support is needed, these studies render it likely that prediction errors scale physically to SD.

A recent study showed that human individuals tend to use model-based approaches when uncertainty in reward increases and that the frontal cortex encodes arbitration between model-based and model-free learning (Lee et al. 2014). However, in the present study participants did not scale prediction errors during the practice sessions, rendering it unlikely that the SD cues functioned as a prior for scaling prediction errors. Participants may, however, have used the practice sessions to construct a model of the degree of reward variability that was used to scale prediction errors during the main task. However, the adaptive models that provided evidence that participants scaled prediction errors updated SD on a trial-by-trial basis. It is thus unlikely that participants solely used a model-based approach to guide prediction error scaling.

These results should be treated with caution, as model comparison using BIC only favored the adaptive Pearce-Hall model after removal of the free parameters used to estimate the initial expected SD of reward. However, all adaptive models were strongly favored with AIC and likelihood ratio tests.

## GRANTS

This work was supported by the Wellcome Trust and the Niels Stensen Foundation.

## DISCLOSURES

No conflicts of interest, financial or otherwise, are declared by the author(s).

## AUTHOR CONTRIBUTIONS

K.M.J.D. and W.S. conception and design of research; K.M.J.D. performed experiments; K.M.J.D. analyzed data; K.M.J.D. and W.S. interpreted results of experiments; K.M.J.D. prepared figures; K.M.J.D. drafted manuscript; K.M.J.D. and W.S. edited and revised manuscript; K.M.J.D. and W.S. approved final version of manuscript.
